# What Pus Is Not

**Published:** 1874-04-01

**Authors:** Lester Curtis


### WHAT PUS IS NOT.

By Lester Curtis, M.D.

A FEW years ago Conheim published some observations on the white blood corpuscle, which confirmed the older observations of Waller and Beale, and called attention to them; for previous to this time they had attracted little notice, especially on the continent of Europe. These observations showed that, in inflammation, many of the white blood corpuscles pass through the walls of the capillaries, and appear outside of them. The corpuscles outside the vessels continue their amoebiform movements, and, possessing the power of locomotion, were called "wandering *cells*." (?)

At the time of these observations it was well known that the fresh pus corpuscle, also, had an amoebiform movement similar to that of the white blood corpuscle. Pus occurs as the result of inflammation; and where there is inflammation there are large numbers of wandering cells. Conheim concluded, therefore, that pus corpuscles came from the wandering cells, and, as the wandering cells came from the white blood corpuscles, therefore, that a pus corpuscle was a white blood corpuscle. He rejected as erroneous the previous opinion that pus could be derived from any other source than the white blood corpuscles.

Conheim's conclusion that the pus corpuscle and the white blood corpuscle are identical, has been widely accepted. It is due partly to the acceptation of this theory that the name "*leucocyte*" has arisen, a name which is applied indiscriminately to the white blood corpuscle, the lymph corpuscle, the wandering cell, and the pus corpuscle. Some, in publishing their acceptation of the theory, have added the saving epithet "*morphologically*" to the "*identical*," evidently implying some doubt, after all, as to its correctness.

In spite, however, of the general acceptation of the opinion, it appears to me to be inconsistent with certain well-known facts. It is my purpose to present some of these facts, and show wherein they are inconsistent with the theory. I shall consider the subject from Conheim's standpoint: supposing that all pus originates from white blood corpuscles, although I consider the proof of such sole origin as far from complete.

In the first place, it by no means follows that, because a pus corpuscle is derived from a white blood corpuscle, it is identical with a white blood corpuscle. The white blood corpuscles are mere stages of growth, just as a chrysalis, or a tadpole, is a stage of growth. They have no particular function of their own, as, for instance, the red corpuscles have; they only exist in order that they may be developed into something else. If this is the case, it is not only supposable that, under the changed conditions of nutrition to which the wandering cells are subjected, outside the vessels, they should undergo a change; but it is difficult to understand how they should continue to be the same that they were within the vessels.

Mere similarity of form and appearance is, as we all know, one of the least reliable of resemblances; and the fact that a pus corpuscle appears to be like a white blood corpuscle can surely go but a short way towards establishing their identity. The sporules of fungi can often be crushed, and the softer, central portion can be freed from the envelope. When this is done, the central portion of the sporule may resemble a white blood corpuscle so closely in every particular, except, perhaps, in size, that even an experienced observer would be unable to distinguish them apart. Would any one, on this account, consider them to be identical? There must be other resemblances between two bodies besides form and appearance merely, to render them identical. They must correspond in all essential particulars; and if they differ in any essential particular, they plainly are not identical. Now let us see if pus corpuscles correspond in all essential particulars with white blood corpuscles.

The white blood corpuscles of every healthy person correspond, in every particular with which we are acquainted, with the white blood corpuscles of every other person; and while there may be, and probably are, points in which the corpuscles of every individual differ from those of every other individual, these differences are so slight that the corpuscles of one person may be substituted for those of another, by transfusion of blood, without disturbance of function. If, then, pus corpuscles are the same things as white blood corpuscles, all pus which has not a specific origin should be similar. I need hardly say, however, that this is notably not the case. No one would suppose for an instant that the pus from an ordinary abscess and that from a purulent ophthalmia were the same. Yet the bland and unirritating pus from the abscess, and the highly contagious pus from the purulent ophthalmia, may have had their origin in a simple, and perhaps similar, irritation; and the white blood corpuscles of the two individuals may preserve their similarity at the same time that the pus shows such great differences. Can things which differ from each other both be similar to the same thing?

Again, the physiological action of pus differs from that of a white blood corpuscle. White blood corpuscles may easily, and with safety, be transferred from the vessels of one individual to those of another; but if pus is injected into the vessels, the result is a serious disturbance. The experiment has been tried of injecting pus into the veins of an animal; a febrile action, dangerous to the life of the animal, is the result; and if some of the blood of this animal is injected into the veins of a second animal, a still severer disturbance than in the first animal is set up. If the blood of the second is injected into the veins of a third, a similar disturbance is set up; and so of a fourth, and so on. The introduction of pus into the veins of the animal has given rise to profound changes in its blood—an effect differing widely from the harmless result of the introduction of the blood corpuscle.

Again, the white blood corpuscles can become organized, and form tissue; or, at least, the wandering cells outside the vessels can become organized; and it is a well-known fact, that from these wandering cells all inflammatory, new formations arise. Some, indeed, maintain that from such wandering cells are produced all the new growth of connective tissue, and all the new formations in the body. Pus, however, cannot become organized, as any one who has observed the mischief done by a small quantity of pus beneath the periosteum of a finger can well appreciate.

If pus, then, originated from a white blood corpuscle, it has lost the power of organizing; and who can tell how great is the difference which has resulted from that loss?

Again, if the pus from our purulent ophthalmia, which may have arisen from a simple irritation, be introduced beneath the lid of a well person, it will, in all probability, set up a disease similar to that in the eye from which it was taken. If a white blood corpuscle had the property of setting up disease, what surgeon would be skillful enough to avoid purulent ophthalmia? The pus from purulent ophthalmia, then, has not only lost the power of organizing, but has acquired noxious properties, which render it hurtful to the person in whom it originated, and dangerous to those with whom it may come in contact. Can any two things differ more widely than the blood corpuscle and this pus—the one a useful and necessary part of the body, and the other a breeder of disease, and an object to be dreaded?

In what I have said, granting what I do not believe, that all pus originates from white blood corpuscles, I have tried to show:

1st. That white blood corpuscles, being in a transition stage, we have no right to expect that, in the changed condition of nutrition to which they are subjected, outside the vessels, they would continue to be the same that they were within the vessels.

2d. That mere similarity of appearance was insufficient evidence of identity.

3d. That different samples of pus are unlike each other; which they would not be if they were white blood corpuscles.

4th. That pus differs from white blood corpuscles:

*a.*—In the disturbance which it sets up when introduced into the vessels.

*b.*—In the loss of the power of organizing.

*c.*—In the frequent acquisition of contagious properties.

These are some, though by no means all, the reasons why I consider that pus is not the same thing as a white blood corpuscle. If I have established the point, it will be something gained; if I have failed, I would esteem it a favor to be shown my error.

---

CASE OF NEURALGIA OF THE TESTES CURED BY ELECTRICITY.—The patient, a young man, free from all syphilitic disease, experienced such intense pain in the testes, that he urgently asked Dr. Felippi to perform castration. The case was carefully made out to be neuralgia, independent of any affection of the testicle or of any accumulation of fæcal matter; and in five sittings the patient was entirely cured. Dr. Felippi made use of a weak and direct constant current.—*L' Imparziale*, No. 16, 1873.

